# Dwarfing and the Underlying Morphological Changes of *Poa alpigena* Plants in Response to Overgrazing Conditions

**DOI:** 10.3390/plants11030336

**Published:** 2022-01-27

**Authors:** Hongxiao Shi, Xinhong Wu, Hai Wang, Sibagen Ha, Tingting Yang, Wenhui Liu

**Affiliations:** 1 Institute of Grassland Research, Chinese Academy of Agricultural Sciences, Hohhot 010010, China; wxh1@vip.sina.com (X.W.); wanghai@caas.cn (H.W.); hasibagen@caas.cn (S.H.); ytt198@126.com (T.Y.); 2 College of Agricultural and Animal Husbandry, Qinghai University, Xining 810016, China

**Keywords:** grazing, *Poa alpigena*, anatomical structure

## Abstract

*Poa alpigena* is a dominant grass species in alpine meadows, which is sensitive to environmental conditions. In this study, we analyzed the characteristics of the anatomical structure of the stems and leaves of *Poa alpigena* in overgrazed and enclosed conditions in order to determine the dwarfing morphological mechanism associated with overgrazing. The results show that leaf thickness, leaf epidermal thickness, epidermal cell area, and phloem thickness increased with increased grazing intensity (*p* < 0.05). In contrast, xylem thickness, mesophyll cell area, and guide wall thickness decreased with an increase in grazing intensity (*p* < 0.05). Mesophyll cell density was relatively unaffected by grazing intensity. Additionally, the plasticity indices of leaf area, upper epidermal cutin layer thickness, and leaf xylem thickness were higher than 0.5. The plasticity indices of stem tube diameter, epidermal cell size, and epidermal cuticle thickness were greater than 0.4. The results of our study indicate that the structural stem and leaf changes in *Poa alpigena* are induced by the water and mechanical stresses that occur under grazing conditions. Thus, plateau plants adapt to grazing stress by increasing the thickness of their leaves, cuticles, and phloem. The mesophyll cell area, as well as the stem epidermal cell area of *Poa alpigena* decreased in response to minor variations in grazing intensity, yet overgrazing did not change its density. However, overgrazing induced a shortening of the leaves and stems, indicating that overgrazing has a dwarfing effect on *Poa alpigena*.

## 1. Introduction

Dwarfing is a common adaptive response of plants to environmental stresses [1,2,3,4,5]. Dwarf grassland plants exhibit several traits, including shortened stature under overgrazing conditions, narrow and short leaves, short internodes, stiff leaves, narrow thickets, and a shallow root distribution [6,7]. Changes to the main plant characteristics and the mechanism regulating the development of the dwarf phenotype of grassland plants have not been fully characterized. Thus, a thorough analysis of the mechanism responsible for the dwarfing of grassland plants is necessary. Alpine plants mainly grow in special climatic conditions such as the alpine and low-temperature conditions on the Qinghai-Tibet Plateau. It is of great ecological significance to study alpine plants under such special climatic conditions [8,9]. In recent years, research studies on the functional ecology of alpine plant anatomy and ultrastructure have been published, mainly focusing on the effects of abiotic stresses such as altitude gradient, light intensity, temperature, and water on the morphological structure of alpine plants [10,11,12]. However, there has been no reporting on the changes in the morphological and anatomical structure of alpine plants under grazing stress.

Plant morphological and anatomical structures are sensitive to environmental stresses. Elucidating the structural changes in plants exposed to environmental stresses may provide important cytological information regarding the mechanism underlying the development of stress-induced damage and reveal how plants can tolerate adverse environments [13]. To date, studies on the effects of environmental factors on plant anatomy have mainly focused on the effects of water, temperature, light, and salt stresses [14,15,16,17]. Additionally, plant anatomical changes in response to ecological conditions in specific habitats have been investigated [18]. The study of environment-induced plant structural changes initially took place in Europe [17,19,20]. Since then, studies have been conducted in various sites in China [14,21,22]. However, there have been relatively few studies on plant morphology and anatomy in the grazing ecosystems of China, especially regarding changes to the dominant species in the alpine meadow grazing ecosystems of the Qinghai-Tibet Plateau under different grazing intensities.

The Qinghai-Tibet Plateau mainly comprises alpine meadows, which are important for maintaining the ecological functions of the region [23]. Determining how alpine meadow plants develop is vital in clarifying how they are damaged by environmental stresses. Research aimed at clarifying the important morphological changes responsible for the dwarfing of *Poa alpigena* plants is warranted. *Poa alpigena* is a gramineous angiosperm that serves as a perennial forage grass species. It is the dominant plant species in the Qinghai-Tibet Plateau’s alpine meadows [24]. Thus, to characterize the mechanism regulating the morphology of *Poa alpigena*, we analyzed changes in the stem and leaf microstructure of *Poa alpigena* affected by compensatory growth and restoration.

## 2. Results

### 2.1. Poa alpigena Stem and Leaf Structures

**Figure 1:**
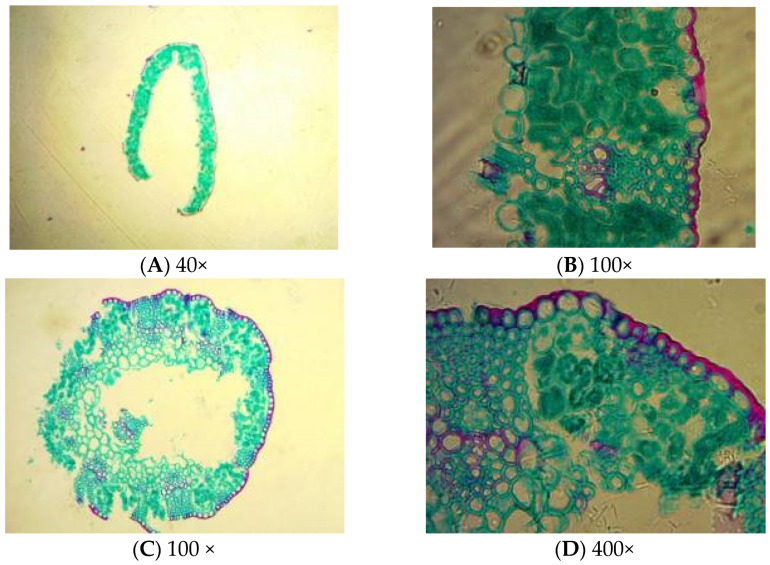
*Poa alpigena* leaf (**A**,**B**) and stem (**C**,**D**) structures.

The *Poa alpigena* epidermal cells were round and tightly packed at the epidermal surface. The mesophyll cells were nearly circular and densely distributed close to the cell wall. Each circular vascular bundle consisted of xylem and phloem (Figure 1A,B).

The stems’ epidermal cuticle consisted of one layer of nearly round, uniform epidermal cells that were tightly packed. The individual circular vascular bundles consisted of xylem and phloem. The basic plant tissue consisted of two or three layers of parenchyma cells just below the epidermis. These parenchyma cells are responsible for the plant’s mechanical organization. A medullary cavity was observed in the middle of the stems (Figure 1C,D).

### 2.2. Effects of Overgrazing and Enclosure on Leaf Structures

#### 2.2.1. Effects of Overgrazing and Enclosure on Leaf Thickness

**Figure 2:**
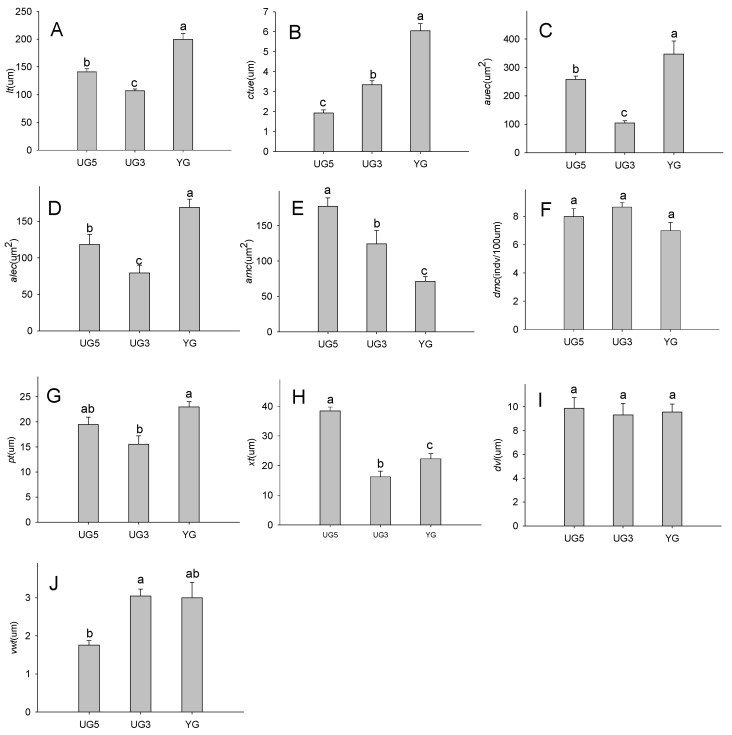
Effects of overgrazing and enclosure on (**A**) leaf thickness, (**B**–**D**) epidermal cuticle, (**E**,**F**) mesophyll cells, (**G**,**H**) phloem and xylem thickness, and (**I**,**J**) vessels. Significant differences are indicated by lowercase letters (*p* < 0.05). Data are presented as the mean ± standard error. Abbreviations: *lt*, leaf thickness; *uect*, upper epidermal cuticle thickness; *auec*, area of the upper epidermal cells; *alec*, area of the lower epidermal cells; *amc*, area of mesophyll cells; *dmc*, density of mesophyll cells; *pt*, phloem thickness; *xt*, xylem thickness; *dvl*, diameter of vessels; *vwt*, vessel wall thickness.

Leaf thickness is somewhat susceptible to the external environment. There were significant differences (*p* < 0.05) in leaf thickness due to grazing intensity, with the YG (heavy grazing) leaves considerably thicker than the UG5 (no grazing from 2009) or UG3 (no grazing from 2011) leaves (YG > UG5 > UG3) (Figure 2A).

#### 2.2.2. Effects of Overgrazing and Enclosure on the Epidermal Cuticle

We observed significant differences in the thickness of the epidermal cuticle (*p* < 0.05), with the samples collected from the overgrazing site being thicker than those collected from the enclosed locations (YG > UG3 > UG5) (Figure 2B). The cell area was significantly larger for the upper epidermal cells than for the lower epidermal cells (*p* < 0.05). Additionally, the area of the upper and lower epidermal cells was larger in the samples collected from the overgrazing site than in the samples collected from the enclosed site (YG > UG5 > UG3) (Figure 2C,D).

#### 2.2.3. Effects of Overgrazing and Enclosure on Mesophyll Cells

There were significant differences in *Poa alpigena* mesophyll cell size among the samples harvested from the overgrazing and the enclosed study locations (*p* < 0.05), with mesophyll cell size increasing as the enclosure duration increased (UG5 > UG3 > YG) (Figure 2E,F). However, grazing type did not significantly affect mesophyll density (*p* > 0.05).

#### 2.2.4. Effects of Overgrazing and Enclosure on Phloem and Xylem Thickness

The phloem of the YG samples was significantly thicker than that of the UG3 samples (*p* < 0.05), while there was no difference between the YG and UG5 samples (*p* > 0.05) (Figure 2G). Additionally, the xylem was significantly thicker in the UG5 samples than in the other collected samples (UG5 > YG > UG3) (*p* < 0.05) (Figure 2H).

#### 2.2.5. Effects of Overgrazing and Enclosure on Vessels

There was no significant difference among study sites regarding vessel diameter (*p* > 0.05) (Figure 2I). In contrast, there were significant differences in the catheter wall thickness between the UG5, UG3, and YG samples (UG3 > YG > UG5) (*p* < 0.05) (Figure 2J).

### 2.3. Effect of Overgrazing and Enclosure on Poa alpigena Stem Structures

#### 2.3.1. Effects of Overgrazing on Stem Epidermis

**Figure 3:**
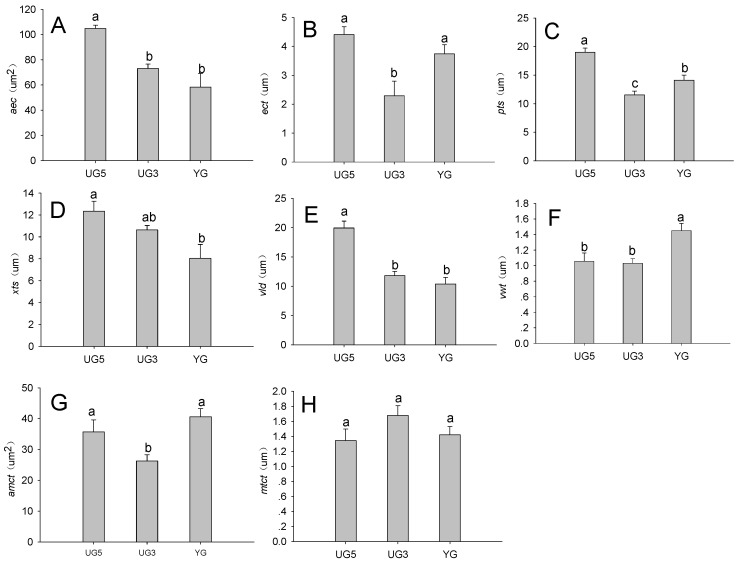
Effects of overgrazing and enclosed on (**A**,**B**) stem epidermis, (**C**,**D**) stem vascular bundle, (**E**,**F**) stem vessel, and (**G**,**H**) stem mechanical tissue. Significant differences are indicated by lowercase letters (*p* < 0.05). Data are presented as the mean ± standard error. Abbreviations: *aec*, area of epidermal cells; *ect*, epidermal cuticle thickness; *pts*, phloem thickness; *xts*, xylem thickness; *vld*, vessel diameter; *vwt*, vessel wall thickness; *amtc*, area of mechanical tissue cells; *mtct*, mechanical tissue cell thickness.

The stem epidermal cell area of the YG and UG3 plants was significantly smaller than that of the UG5 plants (*p* < 0.05) (Figure 3A). Additionally, the stem epidermal cuticle was significantly thicker in the YG and UG5 plants than in the UG3 plants (*p* < 0.05) (Figure 3B).

#### 2.3.2. Effect of Overgrazing on Stem Vascular Bundle Features

There were significant differences (*p* < 0.05) in stem phloem thickness (UG5 > YG > UG3) and xylem thickness (UG5 > UG3 > YG) (Figure 3C,D).

#### 2.3.3. Effects of Overgrazing on Stem Vessel Features

As indicated in Figure 3E, the largest stem vessel diameters were observed in the UG5 samples (*p* < 0.05), while there were no significant differences between the UG3 and YG samples. In contrast, the YG samples had the thickest vessel walls (*p* < 0.05) (Figure 3F), while there were no significant differences between the UG5 and UG3 samples.

#### 2.3.4. Effects of Overgrazing on Stem Mechanical Tissue Features

Our data revealed that the area of mechanical tissue cells was smaller in the UG3 samples than in the UG5 or YG samples (*p* < 0.05) (Figure 3G), while there were no significant differences in the mechanical tissue cell thickness (*p* > 0.05) (Figure 3H).

### 2.4. Plasticity of Leaf and Stem Structures

Relatively high plasticity indices were observed for all leaf measurements (Table 1). The plasticity indices were greater than 0.5 for leaf area, upper epidermal cutin layer thickness, and xylem thickness. The plasticity indices were 0.4–0.5 for leaf thickness and leaf vessel thickness. The lowest plasticity indices (less than 0.4) were observed for leaf phloem thickness and for mesophyll cell and leaf vessel diameters.

In contrast to the plasticity indices of the leaf measurements, those for stem characteristics were relatively low (Table 2). The highest plasticity indices (greater than 0.4) were recorded for stem vessel diameter, epidermal cell size, and epidermal cutin layer thickness. The plasticity indices were 0.3–0.4 for xylem thickness, phloem thickness, and mechanical tissue cell area. The plasticity indices of vessel thickness, parenchymal cell size, and mechanical tissue cell thickness were all less than 0.3.

## 3. Discussion

### 3.1. Effects of Overgrazing and Enclosure on Leaf Structures

Leaves are one of the main organs involved in plants’ important functions (e.g., photosynthesis, respiration, transpiration, and protection), and they are very sensitive to environmental conditions [18]. Thus, it is necessary to analyze *Poa alpigena*’s structural characteristics in response to different grazing conditions.

The leaf is one of the main organs involved in photosynthesis and transpiration [18]. It is also the organ most sensitive to external stimuli [25]. Thus, leaf morphological features can reflect plant adaptations to environmental conditions [26]. Leaf thickness and internal structures change in response to various external stresses [19]. A previous study reported that leaf thickness may be useful as a morphological indicator of environmental effects on plants [27]. In our study, we observed that the leaves were thicker in the samples collected from the open grazing site than in the samples collected from the enclosed grazing locations (*p* < 0.05). From this, we conclude that livestock grazing and trampling induce plants to increase leaf thickness.

Cutin layer thickness is an important indicator of plants’ drought resistance. Plants adapted to drought conditions usually have a well-developed cutin layer [28]. We observed that YG (heavy grazing) samples had thicker leaves and cutin layers than the UG3 (no grazing from 2011) or UG5 (no grazing from 2009) samples (*p* < 0.05), suggesting that plants adapt to the effects of grazing by increasing the thickness of the cutin layer, which can prevent water loss caused by excessive transpiration. One of the consequences of grazing is increased soil density, which results in decreased soil permeability [29]. Increasing the thickness of the cutin layer may improve water use efficiency and decrease the palatability of the plants to grazing animals. Therefore, under overgrazing conditions, *Poa alpigena* thicken their corneous layer to defend against the stress imposed by grazing livestock [30]. The leaf epidermis usually consists of the corneum and one layer of epidermal cells. The epidermal cell traits may reflect the overall plant traits to some extent [31].

Mesophyll cells represent one of the main sites of photosynthesis and respiration activities, and their structural traits directly influence plant growth. Our data suggest that in response to increased grazing, mesophyll cell area tends to decrease (*p* < 0.05), while mesophyll cell density is unaffected (*p* > 0.05). *Poa alpigena* leaves are relatively short under overgrazing conditions. Decreases in mesophyll cell size can minimize transpiration and improve water use efficiency to ensure normal photosynthetic activities [23]. Thus, decreases in mesophyll cell area may be the main factor influencing the dwarfing of *Poa alpigena* plants.

Vascular bundles (i.e., xylem and phloem) are essential for plant growth because of their role in transporting nutrients and water throughout the plant. Phloem thickness increased under overgrazing conditions, while xylem thickness exhibited the opposite trend. The thickening of phloem likely increases the mechanical strength of leaves and may help to protect leaves from grazing livestock under overgrazing conditions. In contrast, decreases in xylem thickness affect leaf transpiration and water use efficiency. Thus, the observed changes in the thickness of *Poa alpigena* leaf phloem and xylem under overgrazing conditions are related to plant adaptations to grazing stress.

The vessels of the water transport system have important functions throughout the plant development period. These vessels are made from the lignified walls of dead cells and are prevalent in the xylem of angiosperms. The vessel diameter affects the efficiency of water conductance, with wider vessels correlated with more efficient water transportation [31]. Additionally, the vessel characteristics influence xylem mechanical strength, and vessel thickness is the main structural feature used as an indicator of the mechanical properties of plants [31]. We did not observe any significant differences in vessel diameter among the collected samples (*p* > 0.05). However, our data indicated that vessel walls were thicker in the YG samples compared to the UG3 and UG5 samples (*p* < 0.05). These results suggest that plants protect themselves from the mechanical damage caused by the trampling and grazing of livestock in overgrazing conditions by increasing the catheter wall thickness.

### 3.2. Effects of Overgrazing on Stem Structures

The stem is vital for transporting water and nutrients throughout a plant [32], and changes to environmental conditions can influence stem structures [31]. Therefore, analyzing *Poa alpigena* stem structures under overgrazing and enclosed conditions is warranted.

A stem’s epidermal cells are in direct contact with the external environment. These cells have a considerable role in regulating the internal conditions of plant tissues, and they help plants to adapt to external stresses. The extent of any changes in the external environment influences the change in the stem’s epidermal cutin layer thickness [31]. In our study, the epidermal cutin layer thickened with increased grazing intensity (*p* < 0.05). This thickening of the cutin layer represents a defense strategy adopted by plants in response to overgrazing. Additionally, the *Poa alpigena* epidermal cell area decreased because of grazing, implying that one of the plant’s adaptive responses involves a shortening of the stem.

Xylem and phloem regulate the water and nutrient use efficiency of plants. Structural changes to the vascular bundles mediate whether plant activities are directed toward survival or growth [31]. The thickness of stem phloem and xylem increased with longer enclosure periods. This suggests that if plants are not required to respond to grazing stress, more resources may be used for growth and development (e.g., increased plant height).

Overgrazing conditions resulted in a significant decrease in stem diameter (*p* < 0.05). Under these conditions, plants regulate their use of limited water resources by inducing changes to the catheter diameter, leading to significant increases in the catheter wall thickness (*p* < 0.05). Thickening of the vessel wall can ensure the long-distance transportation of water. The size and thickness of the mechanical cells in stems tended to increase under overgrazing conditions, which may enhance the mechanical strength of the stem.

### 3.3. Adaptability of Stem and Leaf Structures to Overgrazing

Environmental conditions influence plant morphology and anatomy. Plants adapt to environmental factors via morphological changes [33,34]. We compared the plasticity of *Poa alpigena* stem and leaf structural features. The plasticity indices were higher for leaves than for stems, suggesting that leaves are more susceptible to grazing stress. Regarding the plasticity indices of specific traits, the epidermal cell area, the thickness of the upper epidermis, and leaf xylem thickness were relatively high (i.e., greater than 0.5). Additionally, the plasticity indices were greater than 0.4 for stem vessel diameter, epidermal cell size, and epidermal cutin layer thickness. Our results imply that these traits are sensitive indicators of *Poa alpigena* responses to grazing stress.

## 4. Conclusions

Decreases in mesophyll and stem epidermal cell area caused by overgrazing result in the production of short leaves and stems. This suggests that decreases in mesophyll and stem epidermal cell area are critical in the dwarfing of *Poa alpigena* plants. In addition, under overgrazing conditions, the mesophyll cell area and the stem epidermal cell area decreased, but the density did not change significantly, indicating that the leaves and stems of *Poa alpigena* tend to shorten, resulting in dwarfing. Under overgrazing conditions, the selective feeding behavior of livestock leads to the shortening of tall grasses. Because of the resulting weakening of the shading effect, plants are exposed to increased light intensity. Additionally, livestock trampling increases the grassland soil surface density. This decreases the soil water infiltration rate, which affects plant water absorption. The trampling and grazing of livestock in overgrazing conditions affect the nutrient content and nutrient use efficiency of the plant community. The resulting small changes to plant growth conditions alter plant structures, leading to the dwarfing of individual plants [35]. The observations described in this paper reveal that plants undergo structural changes to adapt to open grazing conditions. This is consistent with the findings of an earlier study [36]. The focus of future research will be whether the reduction in the surface area of mesophyll cells and stem epidermal cells is caused by changes in cell structure or changes in the functions of mitochondria and chloroplasts.

## 5. Materials and Methods

### 5.1. Research Overview

This study was conducted at the Ministry of Agriculture Yushu Alpine Grassland Resource and Ecological Environment at the Key Field Scientific Observation Station (33°24′30″ N, 97°18′00″ E). The study region is part of the Qinghai-Tibet Plateau, with an average altitude of 4250 m above sea level, an annual average temperature of −3.4 °C, and an average annual rainfall of 374.2–721.2 mm. The grassland type is *alpine kobresia* weeds meadow, the constructive species is *Kobresia pygmea*, and the dominant species are *Kobresia kansuensis*, *Stipa Aliena*, *Poa crymophila*, *Elymus natans*, *Potehtilla saundersiana, Anaphalis lactea,* and *Thalictrum alpinum*.

### 5.2. Plot Conditions and Sampling

The following three types of grassland test plots were prepared: whole-year grazing (YG) (i.e., open grazing, 1.74 sheep/ha), fenced for 3 years (UG3), and fenced for 5 years (UG5). Three plots were prepared for each type (i.e., three replicates). The detailed characteristics of the sample are shown in the following table (Table 3).

### 5.3. Plots Sampling

Three plots were prepared for each type (i.e., three replicates), and six *Poa alpigena* adult individuals per replicate were sampled, for a total of 18 individuals. Samples were collected in early August 2013–2015. Stems and leaves were cut into 1–2 cm pieces and then placed in brown bottles containing formalin–acetic acid–alcohol. The bottle openings were filled with a rubber plug, after which the air was extracted until the plant tissues settled at the bottom. The bottles were then preserved at 4 °C until analyzed.

### 5.4. Sample Preparation for Microscopic Analyses

Paraffin sections of plant tissues were prepared to study the root, stem, and leaf cross-sections. The treated materials were washed with water, dehydrated, cleared, dipped in wax, embedded, and manually sliced (10 μm thick). The slices were dried and sealed with neutral gum to prepare permanent sections for microscopic analyses.

### 5.5. Microscopy

A Shineso-MIC microscopic image analysis system was used to obtain images of the prepared plant tissues (40×, 100×, and 400× magnifications). The system software (Shineso-MIC, Hangzhou, China) was used to analyze the stem and leaf epidermal cell size and area, vascular bundles, mechanical tissues, and other related plant parts.

### 5.6. Data Analysis

Data were collected for several leaf structural characteristics, including leaf thickness, upper epidermis thickness, upper epidermal cell area, mesophyll cell size, mesophyll cell density, phloem and xylem thickness, catheter caliber, and catheter wall thickness. Additionally, data were obtained for stem structural features, such as epidermal cuticle thickness, epidermal cell area, phloem and xylem thickness, stem and tube wall thickness, catheter diameter, mechanical cell area, and mechanical cell wall thickness. Differences in the stem and leaf structures among the YG, UG3, and UG5 samples were analyzed by a single-factor analysis of variance that revealed the extent of the influence of overgrazing on anatomical characteristics. The plasticity indices of the leaf and stem structures were calculated with the following formula: PI = 1 − x/X, where x and X refer to the lowest and highest averages for a certain trait, respectively [37]. SPSS 17.0 software was used for statistical analysis and variance analysis, with a 0.05 and 0.01 significance level test. The data were mapped by SigmaPlot 12.0. 

## Figures and Tables

**Table 1:** Plasticity indices of leaf structural characteristics.

*lt*	*uect*	*auec*	*alec*	*amc*	*dmc*	*pt*	*xt*	*dvl*	*vwt*
0.46	0.68	0.53	0.70	0.60	0.19	0.33	0.58	0.06	0.42

Notes: *lt*, leaf thickness; *uect*, upper epidermal cuticle thickness; *auec*, area of the upper epidermal cells; *alec*, area of the lower epidermal cells; *amc*, area of mesophyll cells; *dmc*, density of mesophyll cells; *pt*, phloem thickness; *xt*, xylem thickness; *dvl*, diameter of vessels; *vwt*, vessel wall thickness.

**Table 2:** Plasticity indices of stem structural characteristics.

*vld*	*vwt*	*aec*	*ect*	*xts*	*pts*	*mtct*	*amtc*
0.41	0.29	0.44	0.48	0.35	0.39	0.20	0.35

Notes: *vld*, vessel diameter; *vwt*, vessel wall thickness; *aec*, area of epidermal cells; *ect*, epidermal cuticle thickness; *xts*, xylem thickness; *pts*, phloem thickness; *mtct*, mechanical tissue cell thickness; *amtc*, area of mechanical tissue cells.

**Table 3:** The basic characteristics of the test plots.

Plot	Latitude			Grazing Intensity		Land-Use-Type	Dominant Species
	Longitude	Latitude	Altitude (m)		Area (ha)		
YG	33°0′27.72″	97°18′13.32″	4279.9	Heavy grazing	66.67	Annual grazing from January to December	*Kobresia pygmea*, *Kobresia kansuensis*
UG3	33°21′38.25″	97°18′6.65″	4277.4	No grazing	6.67	Fenced and no grazing in 2011	*Elymus natans*, *Poa crymophila*, *Kobresia pygmea*, *Kobresia kansuensis*
UG5	33°21′30.24″	97°18′10.08″	4198.1	No grazing	13.33	Fenced and no grazing in 2009	*Elymus natans*, *Poa crymophila*, *Kobresia pygmea*

## Data Availability

The data presented in this study are available within the article.
